# Multiple myeloma associated with an Evan’s syndrome

**DOI:** 10.11604/pamj.2016.25.127.10750

**Published:** 2016-11-01

**Authors:** Achour Bechir, Regaieg Haifa, Ben Sayed Nesrine, Bouslema Emna, Mejdoub Senda, Achour Asma, Bouatay Bouzouita Amina, Senda Mrabet, Ben Youssef Yosra, Kortas Mondher, Khelif Abderrahim

**Affiliations:** 1 Department of Hematology, Farhat Hached Hospital, Sousse Tunisia; 2 Department of Radiology, Farhat Hached Hospital, Sousse Tunisia; 3 Laboratory of biologic Hematology, Farhat Hached Hospital, Sousse Tunisia; 4 Departement of Nephrology, Sahloul Hospital, Sousse Tunisia

**Keywords:** Multiple myeloma, evan´s syndrome, autoimmun hemolytic anemia, thrombocytopenia

## Abstract

Auto-immun events are rare in multiple myeloma (MM). Here, we report one MM case complicated by Evans syndrome (Autoimmun hemolytic anemia (AIHA) associated with thrombocytopenia). A 52-year-old man was admitted in nephrology department with severe anemia, renal insufficiency and hypergamma globulinemia. Laboratory exams showed acute hemolysis due to an IgG warm autoantibody. Serum electrophoresis revealed the presence of a monoclonal IgG protein and urinary M protein was 2g/day. A whole body CT-Scan showed osteolytic lesions of vertebral body of C5, D4, L3, L4 and the left iliac wing. The diagnosis of multiple myeloma and Evan's syndrome was made, we underwent chemotherapy by BTD (bortezomib-thalidomide-dexamethasone) and continuous corticosteroid therapy but unfortunately the patient died secondary of a Lactic acidosis. The relationship between MM and hemolysis remain unclear.

## Introduction

Multiple myeloma (MM) is a B-cell malignancy characterized by monoclonal proliferation of plasma cells in the bone marrow. Anemia associated with MM has been reported to arise from disorder of maturation of erythroid lineage, shortening life span of erythrocytes and iron deficiency associated with increased tumor cell mass [1, 2], while auto immune hemolytic anemia (AHAI) has been rarely reported. The association between AIHA and MM remained unclear [3]. Here, we report a second case of MM complicated with Evan's syndrome.

## Patient and observation

**Figure 1:**
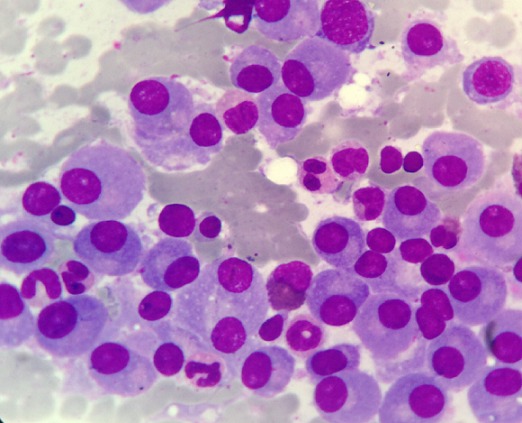
Multiple myeloma– cytology: magnification x 100: diffuse infiltration of the bone marrow aspiration by mature plasma cells. Most cells display mature chromatin, low nuclear cytoplasmic ratio and flamed cytoplasm

**Figure 2:**
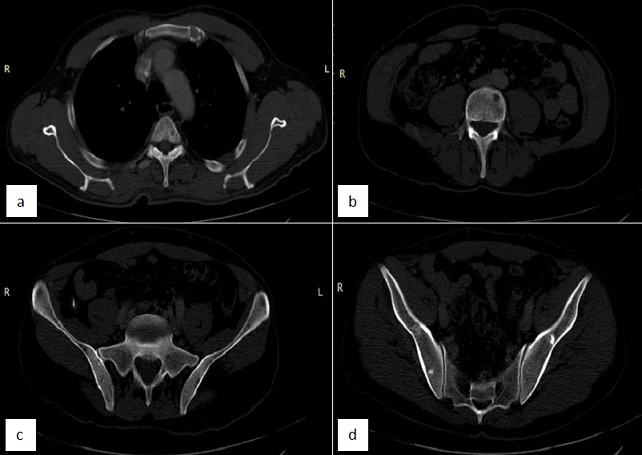
Cervical-thoracic-lumbo-sacral and pelvis CT scan: (A, B, C, D) diffuse lytic lesions and sclerotic bony lesions in iliac bones

A 52 year's old men with no medical history was admitted in the department of nephrology for 2 months of fatigue, dizziness and acute renal failure: creatinine 330 µmol/l and clearance of creat 14 ml /min. No abnormalities were observed in physical examination except conjunctival jaundice, liver and spleen were not palpable. Laboratory investigation showed: hemoglobin 86g/L, red blood cell (RBC) 3.67*1012/L, reticulocytes count of 5.2%, white blood cells (WBC) 12.3*109/L, platelet count 36*109/L, lactate dehydrogenase (LDH) was 451UI/L, indirect bilirubin 49 mmol/L and total bilirubin 96 mmol/L. No hypercalemia was noted. Direct Coomb's test (DCT) was positive for auto-antibody. The autoantibody eluted was warm IgG antibody. At the same time, total serum protein 113 g/L and a spike in gamma globulin estimated to 69 g/l. Serum immune electrophoresis showed that the M protein was the IgG kappa chain. Urinary M protein was 2g/day. Bone marrow aspirates showed infiltration by 78% of plasma cells (Figure 1). Cytogenetics by conventional karyotyping showed no chromosomal abnormalities. A whole body CT-Scan showed osteolytic lesions of vertebral body of C5, D4, L3, L4 and the left iliac wing (Figure 2). After a diagnosis of multiple myeloma and Evan's syndrome was made, chemotherapy was rapidly initiated by BTD (bortezomib-thalidomide-dexamethasone) and continuous corticosteroid therapy. Evolution was marked by deterioration of the hematological and renal state along with the occurrence of hemorrhagic symptoms such as echymosis and haematemesis. The patient died secondary of a lactic acidosis.

## Discussion

The most common complications of multiple myeloma include anemia, hypercalcemia, pathologic fractures, renal failure and recurrent infections. More than two thirds of all patients with MM have anemia [4, 5]. Anemia occurring in patients with MM is multifactorial. Several factors have been implicated in the pathogenesis : Bone marrow (BM) infiltration by malignant plasma cells, hemodilution linked to the hyperprotidemia, Chemotherapy-induced bone marrow suppression, iron deficiency and vitamins, relative erythropoietin (EPO) deficiency (due partly to renal impairment) [6].

A rare cause of anemia is hemolysis due to auto immune, drug induced and traumatic-microangiopathic process or flowing intensive chemotherapy [7]. In fact auto-immunity is a current manifestation in other hematological malignancies such as chronic lymphocytic leukemia or non Hodgkin lymphomas and it is extremely rare in MM. Previous studies showed that only about 4% of AIHA patients had myeloma [8]. While others reports have stated that myeloma is rarely associated with hemolytic anemia [8]. Thrombocytopenia is seen frequently in patients with multiple myeloma when most often the etiology is either marrow replacement by the myeloma cells or treatment with anti-myeloma agents. Immune thrombocytopenia is often seen in patients with lymphoproliferative disorders such as non-Hodgkin´s lymphoma and chronic lymphocytic leukaemia. However this is has only rarely been documented in patients with multiple myeloma [9]. In addition, ITP can be associated with AIHA as Evans´ syndrome, with hemolysis usually preceding thrombocytopenia [10]. Eight cases of AIHA and one case of Evan's syndrome have been described in patients with MM, in none of these cases the pathogenesis of hemolysis was lucid [5, 11].

In our patient the diagnosis of MM was clear, both M-protein produced by MM and the immonoglobin that bound to the patient red cell's was IgG type which suggest that the M-protein may has been responsible of the hemolysis, however this could not be confirmed and it needs further investigations. It's unclear wether auto-immune manifestations are results of MM or it's just a coincidence to be present in the same time. Only one study lead by Wada and al showed that M-protein and the Ig directed against red cells was from the same subtype and subtype IgG1kappa [8].

Also it has been suggested that MM is a B-cell malignancy associated with a marked immune disturbance that may allow normally suppressed clones to develop and produce auto antibodies against red cell surface antigens [12]. Counter selection of VH4-36 segment in MM due to self-tolerance mechanism. Several drugs had also been shown to be causative agent for immune mediated hemolysis in MM such as lenalidomide and interfern α due to immunomodelatery effects [13, 14]. The fast awful outcome of the patient assume that was probably a refractory form of MM associated to Evan's syndrome, as well as Hofer S et al reported the effectiveness of Rituximab in refractory AIHA and MM [15]. So we should have tried the anti CD20 monoclonal antibodies to resolve the situation.

## Conclusion

Although auto-immune events occurs rarely in patients with MM, they must be kept in mind. Further studies are necessary to understand the mechanism of hemolysis in this disease.
